# Patterns of motor activity in spontaneously hypertensive rats compared to Wistar Kyoto rats

**DOI:** 10.1186/s12993-016-0117-9

**Published:** 2016-12-01

**Authors:** Ole Bernt Fasmer, Espen Borgå Johansen

**Affiliations:** 1 Department of Clinical Medicine, Section for Psychiatry, Faculty of Medicine and Dentistry, University of Bergen, Bergen, Norway; 2 Division of Psychiatry, Haukeland University Hospital, Bergen, Norway; 3 K.G. Jebsen Centre for Research on Neuropsychiatric Disorders, Bergen, Norway; 4 Oslo and Akershus University College, Stensberggata 26, 0170 Oslo, Norway

**Keywords:** ADHD, SHR, WKY, Behavioral variability, Motor activity, Video-analyses

## Abstract

**Background:**

Increased motor activity is a defining characteristic of patients with ADHD, and spontaneously hypertensive rats have been suggested to be an animal model of this disorder. In the present study, we wanted to use linear and non-linear methods to explore differences in motor activity patterns in SHR/NCrl rats compared to Wistar Kyoto (WKY/NHsd) rats.

**Methods:**

A total number of 42 rats (23 SHR/NCrl and 19 WKY/NHsd, male and female) were tested. At PND 51, the animals’ movements were video-recorded during an operant test procedure that lasted 90 min. Total activity level and velocity (mean and maximum), standard deviation (SD) and root mean square successive differences (RMSSD) were calculated. In addition, we used Fourier analysis, autocorrelations and two measures of complexity to characterize the time series; sample entropy and symbolic dynamics.

**Results:**

The SHR/NCrl rats showed increased total activity levels in addition to increased mean and maximum velocity of movements. The variability measures, SD and RMSSD, were markedly lower in the SHR/NCrl compared to the WKY/NHsd rats. At the same time, the SHR/NCrl rats displayed a higher complexity of the time series, particularly with regard to the total activity level as evidenced by analyses of sample entropy and symbolic dynamics. Autocorrelation analyses also showed differences between the two strains. In the Fourier analysis, the SHR/NCrl rats had an increased variance in the high frequency part of the spectrum, corresponding to the time period of 9–17 s.

**Conclusion:**

The findings show that in addition to increased total activity and velocity of movement, the organization of behavior is different in SHR/NCrl relative to WKY/NHsd controls. Compared to controls, behavioral variability is reduced in SHR/NCrl at an aggregate level, and, concomitantly, more complex and unpredictable from moment-to-moment. These finding emphasize the importance of the measures and methods used when characterizing behavioral variability. If valid for ADHD, the results indicate that decreased behavioral variability can co-exist with increased behavioral complexity, thus representing a challenge to current theories of variability in ADHD.

## Background

Increased motor activity is a defining characteristic of patients with attention-deficit/hyperactivity disorder (ADHD), combined and hyperactive subgroups. This is based on observations of children with ADHD and on objective registrations with actigraphs [1]. Studies of reaction times, as well as other behavioral measures in patients with ADHD, have repeatedly shown increased intraindividual variability (IIV) as a characteristic feature of ADHD [2–18].

Spontaneously hypertensive (SHR/NCrl) rats have been suggested to be an animal model of ADHD [19], and in several test paradigms display behavior similar to that seen in patients with ADHD, including increased motor activity, impulsivity, and inattention. Another similar feature observed in the behavior of SHR/NCrl is increased IIV [20–24]. However, there are divergent views on SHR/NCrl as a valid model of ADHD [25] which may possibly be related to the control strain used in the experiments [26].

A characteristic feature of different disorders or disease processes may be increased order and regularity of behavior, i.e. reduced complexity [27, 28]. Biological systems can seldom be fully characterized by simple linear processes, and additional mathematical methods are required obtained from the field of non-linear system, complexity theory and chaos theory [29]. At a molar, aggregated level, behavioral variability is quantitatively described by measures such as standard deviation and root mean square of successive differences. However, such measures do not capture behavior variability at a local, molecular, moment-to-moment level [30]. Therefore, non-linear methods, such as different measures of complexity and entropy, have in recent years been employed to analyze biological time series. Such methods may give additional information to that obtained by traditional linear methods, and can be used to identify the underlying neural mechanisms of the system being studied.

In the present report, we have analyzed video-recorded motor behavior of SHR/NCrl rats in order to look for differences in behavioral organization between this strain and control rats of the WKY/NHsd strain. In addition to total activity levels and velocity of movement, we have used both linear and non-linear methods to analyze movement patterns. We have used standard deviation (SD) and root mean square successive differences (RMSSD) to indicate the molar, overall level of variability. These measures have been used in the study of reaction time variability in ADHD patients [31] and also in the study of motor activity of psychiatric patients assessed with actigraphs [32–34]. For analyses of molecular behavioral variability, we included analyses of autocorrelations, which have been used to assess response variability in children with ADHD [2, 3]. Additionally, to investigate variability in different frequency domains, we have employed Fourier analyses, which is a well-established method in many different fields, and which have been used together with SD and RMSSD in studies of motor activity [32–34]. To obtain a measure of complexity we have used sample entropy and symbolic dynamics which are two methods that tolerate a reasonable degree of noise (as usually is the case with biological systems). Both methods were used in the actigraph-studies mentioned above [32–34] and also in the study of reaction time variability in ADHD patients where increased variability and reduced complexity were found [31].

Reduced behavioral complexity is suggested to be a characteristic of different disorders or disease processes, and several studies show that intraindividual variability is increased in ADHD as well as in SHR/NCrl. Thus, our hypothesis when conducting this study was that the behavior of SHR/NCrl rats would be characterized by increased variability and reduced complexity compared to WKY/NHsd rats, both with regard to total activity and velocity of movement.

## Methods

### Subjects

A total number of 42 animals, 23 SHR/NCrl rats (11 females and 12 males) and 19 WKY/NHsd rats (11 females and 8 males) participated in the present experiment. The rats were primarily employed as controls in a behavioral study on the effects of polychlorinated biphenyl 153 in a rat model of ADHD, and had been orally administered corn oil at postnatal days (PND) 8, 14, and 20 [35]. Data from PND 51 were used in the present analyses. The study was approved by the Norwegian Animal Research Authority (NARA) (project id. no. 590), and conducted in accordance with the laws and regulations controlling experiments on live animals in Norway.

### Apparatus and behavioral procedure

Details of the apparatus and experimental procedure are described in [35, 36]. In brief, 16 Campden Instruments operant chambers enclosed in sound-resistant outer housings were used in the current study. The animal’s working space was 25 × 25 × 25 (height) in half of the chambers, and 25 × 25 × 20 (height) in the other half. Each chamber was equipped with two levers, one positioned on each side of a small, recessed cubicle where reinforcers (water) were delivered contingent on lever-pressing.

A variable interval 180 s schedule of reinforcement was in effect for the session analyzed in the present study and for the 17 prior sessions. A cue light was located above each lever, and only presses on the lever signaled by light produced reinforcers. Then, the cue light above the other lever was off, and pressing this lever had no consequences. Following each reinforcer delivery, the reinforcer-producing lever randomly switched side. The behavioral procedure has been described as a simultaneous visual discrimination task [36].

Behavior was recorded by a video camera manufactured by Tracer Technology Co., Ltd, Taiwan (Mini Color Hidden Cameras, 420TVL, 0,1 lux) mounted in the upper rear corner of the ceiling. The camera was controlled by the VR Live Capture computer program (Novus Security, Warsaw, Poland) saving video-files (15 frames/s) for analyses.

### Video recordings

The animals were video-recorded during the whole 90-min session, and frame-to-frame analyses of changes in pixels were performed using a computer program developed by Jensenius [37]. Changes in pixels occurred whenever the animal moved, and the total number of pixel-changes was used to quantitate the animal’s locomotion [38]: Total motor activity was calculated as the sum of all pixels that changed from frame to frame divided by the total number of pixels in the video image. The center of the active pixels was used to estimate the animal’s position and calculate velocity (i.e. velocity = change in position/time). For the present analyzes, seven recordings per second were used to calculate total amount of movement and velocity (mean and maximum).

### Data analysis

The first 84 min of each session were used for the analysis of motor activity, either analyzed as one continuous period or divided into three separate periods of 28 min each. Data were analyzed using SPSS 18. Differences between SHR/NCrl and WKY/NHsd rats were compared using *t* tests except for the autocorrelations that were analyzed by way of ANOVA using Statistica 12.

Several different measures of variability were calculated and analyzed in order to characterize behavioral variability at a molar level as well as at a local level. For analyses of behavioral variability at a molar level, mean values, SD, and the RMSSD were used. Additionally, we used four other measures to characterize these motor patterns at a local, fine-grained level; sample entropy, symbolic dynamics, Fourier analysis and autocorrelations.

#### Standard deviation and root mean square successive differences

Each of the three 28-min periods obtained when dividing the first 84 min of the test session in three equal parts contained 130 data points. Each of these points thus encompasses data from a time period of 12.9 s, and represent respectively the total amount of motion, the average (mean) velocity or the maximum velocity during this time period. Standard deviation (SD) and RMSSD were both expressed as percent of the mean.

#### Sample entropy

Sample entropy (http://www.physionet.org) is a nonlinear measure developed to compute the regularity of heart rate and other time series [32, 39–41]. Sample entropy is the negative natural logarithm of an estimate of the conditional probability that two sequences that are similar for *m* points, within a tolerance, remain similar at the next point. Data were normalized before analysis. According to Richman and Moorman [41], we chose the following parameters: *m* = 2 and *r* = 0.2. Time periods of 12.9 s were used for the sample entropy analyses. Sample entropy was calculated using a program downloaded from the web-site PhysioNet, a resource site for the analysis of physiological signals (http://www.physionet.org). This program calculates the sample entropy of time series given in a text format input-file.

#### Symbolic dynamics

The same time series as used for the sample entropy analyses were employed to analyze symbolic dynamics (time periods of 12.9 s). The time series were transformed into series of symbols according to the method described by [42, 43]. For each sequence analyzed, the difference between the maximum and minimum value was divided into 6 equal portions (1–6) and each value of the series was assigned a number from 1 to 6, such that the transformed time series consisted of a string of numbers from 1 to 6. The series were then divided into overlapping sequences of three consecutive numbers. Each sequence was assigned one of four symbols according to the following rule: (1) 0 V—a pattern with no variation (e.g. pattern 333 or 555), (2) 1 V—a pattern with only one variation where two consecutive symbols are equal and the remaining symbol is different (e.g. 522 or 331), (3) 2LV—a pattern with two like variations, such that the 3 symbols ascend or descend (e.g., 641 or 235), and, (4) 2UV—a pattern with two unlike variations (both ascending and descending, e.g., 312 or 451). The occurrence of these four patterns (0, 1 V, 2LV, 2UV) were counted and the results presented as the percentage of the total number of sequences analyzed (n = 129). The symbolic dynamic analyses give an indication of the complexity of the time series.

#### Fourier analysis

For the Fourier analyses (http://www.physionet.org), the first 84 min of the test session were divided into three equal parts, each containing 390 data points, and the middle 256 points from these time series were used. Each data point thus represents a time period of 4.3 s. The reason for using 256 data points is that the Fourier analysis requires series with a length that represents a power of 2 (64, 128, 256). Data were normalized before analysis and no windows were applied. Results are presented as the relation between variance in the high frequency part of the spectrum, 0.116–0.0581 Hz, corresponding to the period 9–17 s, and the low frequency part, 0.0581–0.00091 Hz, corresponding to 17–1100 s.

#### Autocorrelations

The first 84 min of the session were divided into three 28-min sequences, and serial correlations (autocorrelations) of movement and velocity were calculated for each of the three sequences thus expressing the predictability or variability of behavior within a sequence of observations. A total of 42 lags were calculated, where the correlation between e.g. movement at time t and movement at time t + 1 represents lag 1, the correlation between movement at time t and movement at time t + 2 represents lag 2, and so forth. The autocorrelations were calculated for seven recordings of movement or velocity per second. Thus, the 42 lags represent a time period of approximately 6 s.

## Results

### Total motor activity

The SHR/NCrl rats showed substantially higher total motor activity than the WKY/NHsd rats during all three sequences of the test session; 437, 542 and 426% of the activity of the WKY/NHsd rats (Table 1).

**Table 1:** Total amount of motor activity

	WKY	SHR	WKY	SHR	WKY	SHR
	0–28 min		28–56 min		56–84 min	
Mean	671 ± 256	2935 ± 919***	424 ± 205	2298 ± 818***	482 ± 259	2054 ± 658***
SD	136 ± 72	71 ± 33 **	125 ± 33	66 ± 19***	124 ± 31	66 ± 11***
RMSSD	159 ± 91	87 ± 43**	163 ± 41	83 ± 26***	162 ± 40	86 ± 16***
Sample entropy	1.25 ± 0.65	1.87 ± 0.53**	1.32 ± 0.40	1.99 ± 0.40***	1.27 ± 0.46	2.08 ± 0.36**
Fourier analysis	0.56 ± 0.19	0.65 ± 0.17	0.62 ± 0.18	0.70 ± 0.21	0.63 ± 0.22	0.77 ± 0.26
Symbolic dynamics						
0 V	0.6 ± 1.8	0.0 ± 0.0	3.0 ± 3.4	0.0 ± 0.0**	3.7 ± 4.8	0.0 ± 0.2**
1 V	3.7 ± 3.0	0.1 ± 0.4***	10.1 ± 6.0	0.2 ± 0.5***	9.9 ± 6.1	0.6 ± 1.0***
2LV	33.8 ± 3.4	35.5 ± 3.6	28.6 ± 4.8	36.0 ± 4.7***	30.0 ± 5.0	34.4 ± 5.7*
2UV	61.9 ± 3.7	64.4 ± 3.6*	58.3 ± 7.6	63.8 ± 4.5**	56.5 ± 8.0	64.9 ± 5.4***

Activity was analyzed using time periods of 12.9 s. SD and RMSSD are given as % of the mean. For the Fourier analysis results are presented as variance in the high frequency range divided by the variance in the low frequency range. All data are given as mean ± SD

*t* tests: * p < 0.05, ** p < 0.01, *** p < 0.001

At a molar level, both the SD and the RMSSD measures showed reduced behavioral variability in the SHR/NCrl rats. In the three sequences, the SDs in SHR/NCrl were 52, 53 and 53% and the RMSSDs were 55, 51 and 53% of the corresponding values for the WKY/NHsd. Calculating variability for total motor activity without correcting for mean values (using absolute SD values) showed higher variability for SHR/NCrl compared to WKY/NHsd rats, with values that were 214, 284 and 275% of the corresponding values for the WKY/NHsd rats in the three sequences. For RMSSD, the absolute values were also higher for SHR/NCrl compared to WKY/NHsd rats. The values were 226, 279 and 271% of the corresponding values for the WKY/NHsd rats in the three sequences (Table 4).

**Fig. 1:**
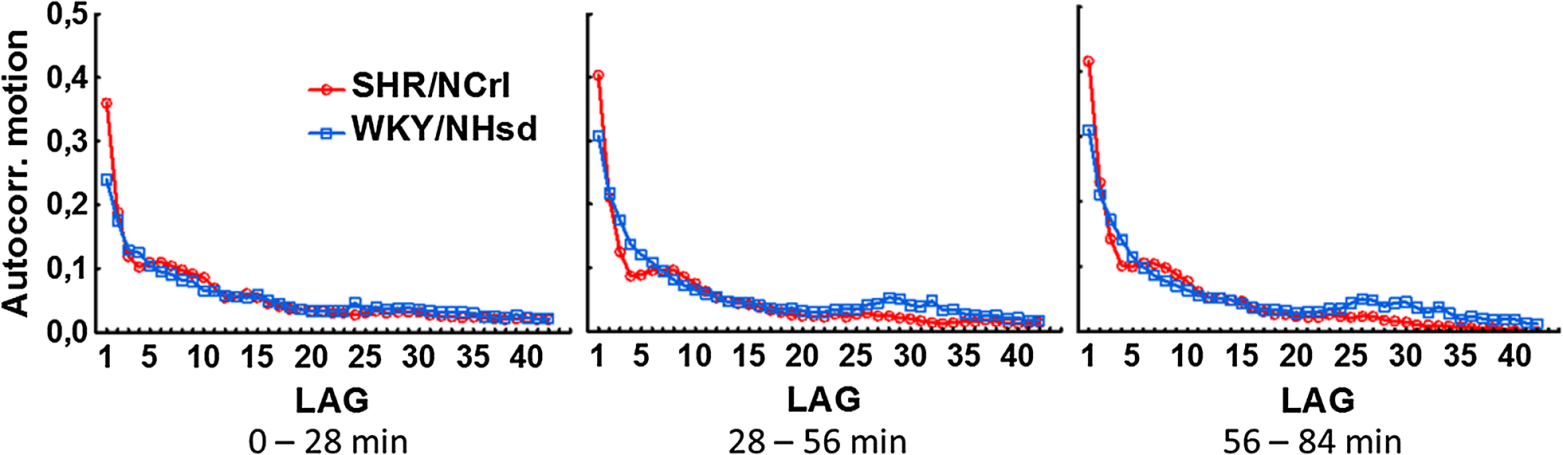
Autocorrelations (lags 1–42) of total motor activity for SHR/NCrl and WKY/NHsd for three 28-min periods representing the first 84 min of the 90-min session

At a molecular level, the Fourier analysis showed that the SHR/NCrl rats had an increased ratio of variance in the high frequency range compared to the low frequency range (16, 13 and 22% higher than the WKY/NHsd rats), but these differences were not significant. The sample entropy was for the SHR/NCrl rats increased to 150, 151 and 164% of the corresponding values for the WKY/NHsd rats in the three sequences. The symbolic dynamic analyses showed that the SHR/NCrl rats had lower values for the 0 and 1 V measures, particularly in the second and third sequences, and correspondingly higher values for 2LV and 2UV. Analyses of motor activity autocorrelations (Fig. 1) showed no statistically significant main effects of strain for the three sequences analyzed. However, statistically significant strain x lag interaction effects were found in all three sequences (0–28, 28–56 and 56–84 min): F (41, 1640) = 3.59; p < 0.0001, F (41, 1640) = 6.68; p < 0.0001, and F (41, 1640) = 7.94; p < 0.0001, respectively. Newman-Keuls post hoc analyses of these significant effects showed that the autocorrelation for lag 1 was higher in SHR/NCrl than in WKY/NHsd controls in all the three sequences, were lower for lags 3–5 in the second sequence, and higher for lags 3–4 in the third sequence (*p*s < 0.05).

### Velocity

**Fig. 2:**
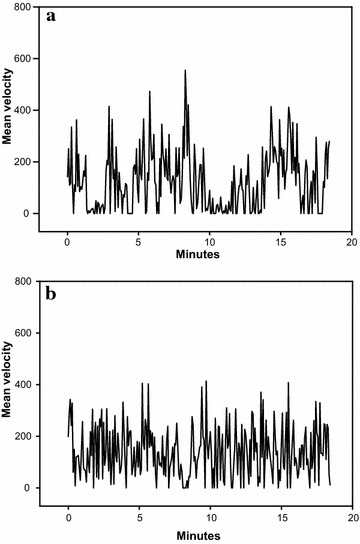
Mean velocity for one SHR/NCrl (**a**) and one WKY/NHsd rat (**b**) during the third 28-min time period of the 90-min session (the last 6 min excluded). The mean velocity during this period was 198 ± 32 (SEM) for all SHR/NCrl and 107 ± 25 (SEM) for all WKY/NHsd, respectively (see Table 2)

The mean velocities of the SHR/NCrl were also significantly higher than in the WKY/NHsd rats, but the differences were smaller than for the motor activity. Mean velocity for the SHR/NCrl rats were 164, 195 and 185% of the corresponding values for the WKY/NHsd rats in the three test sequences (Table 2). This difference is illustrated in Fig. 2 showing mean velocity over time during the third sequence for one SHR/NCrl and one WKY/NHsd rat.

**Table 2:** Mean velocity

	WKY	SHR	WKY	SHR	WKY	SHR
	0–28 min		28–56 min		56–84 min	
Mean	146 ± 20	240 ± 37***	110 ± 24	214 ± 36***	107 ± 25	198 ± 32***
SD	51 ± 9	32 ± 4***	65 ± 10	35 ± 4***	66 ± 13	37 ± 7***
RMSSD	59 ± 10	39 ± 5***	80 ± 16	45 ± 7***	81 ± 16	49 ± 10***
Sample entropy	2.01 ± 0.24	2.20 ± 0.36	2.14 ± 0.33	2.15 ± 0.25	2.05 ± 0.4	2.25 ± 0.28
Fourier analysis	0.52 ± 0.10	0.68 ± 0.20**	0.42 ± 0.09	0.71 ± 0.16***	0.43 ± 0.10	0.76 ± 0.22***
Symbolic dynamics						
0 V	0.1 ± 0.3	0.0 ± 0.0	0.3 ± 0.8	0.0 ± 0.0	1.3 ± 3.5	0.0 ± 0.0
1 V	2.3 ± 2.1	1.0 ± 1.2*	3.0 ± 3.1	1.3 ± 1.0*	3.9 ± 4.6	2.1 ± 1.6
2LV	33.6 ± 4.0	34.9 ± 4.7	34.2 ± 5.1	34.9 ± 4.1	35.0 ± 4.6	32.8 ± 3.9
2UV	64.1 ± 4.1	63.8 ± 4.1	62.5 ± 5.0	63.8 ± 4.3	59.8 ± 6.2	65.1 ± 3.8**

Mean velocity was analyzed using time periods of 12.9 s. SD and RMSSD are given as % of the mean. For the Fourier analysis results are presented as variance in the high frequency range divided by the variance in the low frequency range. All data are given as mean ± SD

*t* tests: * p < 0.05, ** p < 0.01, *** p < 0.001

At a molar level, and similar to the findings for the motor activity, both the SD and the RMSSD measures showed lower variability in the SHR/NCrl rats. In the three test sequences, the SDs of the SHR/NCrl rats were 63, 54 and 56% of the values for the WKY/NHsd rats, and 66, 56 and 60% for the RMSSDs. Calculating variability without correcting for mean values (using absolute SD values) showed higher variability for SHR/NCrl compared to WKY/NHsd rats, with SDs that were 103, 106 and 104% of the corresponding values for the WKY/NHsd rats in the three sequences. For RMSSD, the absolute values were also higher for SHR/NCrl compared to WKY/NHsd rats with values that were 108, 111 and 113% of the corresponding values for the WKY/NHsd rats in the three sequences (Table 4).

**Fig. 3:**
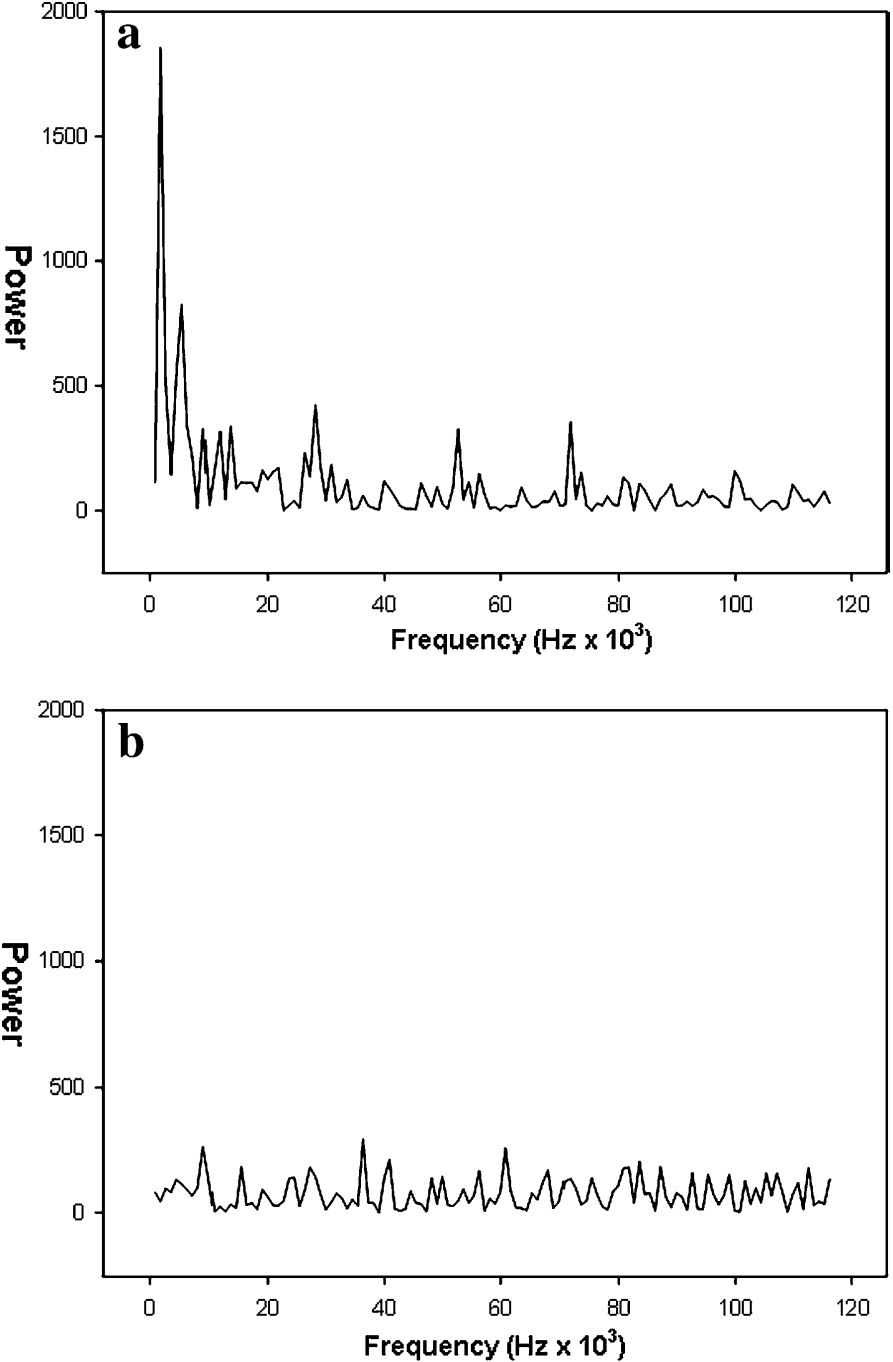
Fourier analysis of the mean velocity data for the SHR/NCrl (**a**) and WKY/NHsd rats (**b**) displayed in Fig. 2. Power spectral density (ordinate) is shown as a function of frequency (*abscissa*), and illustrates the difference in the ratio of variance in the high frequency as compared to the low frequency end of the spectrum between the two strains

**Fig. 4:**
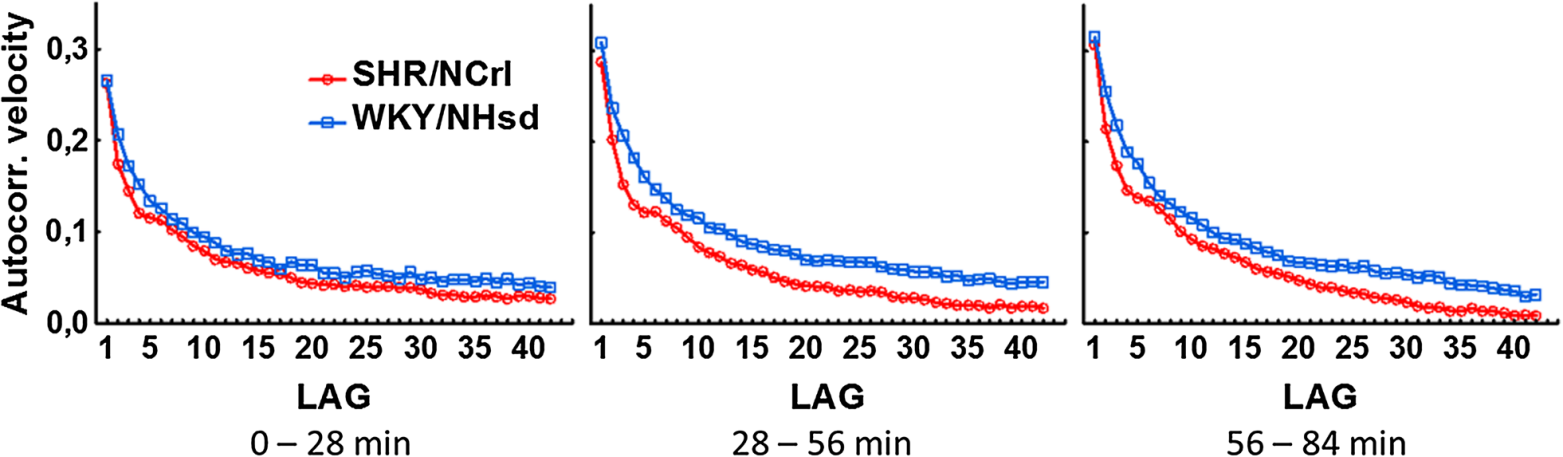
Autocorrelations (lags 1–42) of velocity for SHR/NCrl and WKY/NHsd for three 28-min periods representing the first 84 min of the 90-min session

At a molecular level, the Fourier analysis showed that the SHR/NCrl rats had a significantly increased ratio of variance in the high frequency range compared to the low frequency range. In SHR/NCrl, this ratio was found to be 31, 69 and 77% higher than for the WKY/NHsd rats in the three sequences. As an illustration, Fig. 3 shows the Fourier analysis results during the third sequence for the same animals as in Fig. 2. Contrary to the findings for motor activity, the sample entropy values did not differ between the SHR/NCrl and the WKY/NHsd rats. The symbolic dynamic analyses showed lower values in SHR/NCrl for the 0 and 1 V measures, but only significantly different from WKY/NHsd for 1 V in the first and second sequences, and significantly higher values in SHR/NCrl for 2UV in the third sequence. Further, the analyses showed that autocorrelations of velocity (Fig. 4) were lower in SHR/NCrl than in WKY/NHsd controls in all three sequences (0–28, 28–56 and 56–84 min): F (1, 40) = 5.69; p < 0.05, F (1, 40) = 17.11; p < 0.001, and F (1, 40) = 8.97; p < 0.01, respectively. The analyses also showed a statistically significant strain × lag interaction effect during the third sequence, F (41, 1640) = 1.84; p < 0.001. Newman-Keuls post hoc tests showed that autocorrelations were lower in SHR/NCrl than in WKY/NHsd for lags 2–5 (*ps* < 0.05*)*.

The maximum velocities of the SHR/NCrl rats were significantly higher than those of the WKY/NHsd rats, but the differences were smaller than for the mean velocity. The values for the SHR/NCrl rats were 12, 23 and 22% higher than the corresponding values for the WKY/NHsd rats in the three test sequences (Table 3). Similar to the findings for the motor activity and the mean velocity, both the SD and the RMSSD measures showed lower variability in the SHR/NCrl rats. In the three test sequences, the SDs for the SHR/NCrl rats were 67, 55 and 59% of the values for the WKY/NHsd rats, whereas the corresponding values for the RMSSDs were 69, 61 and 63%. Calculating variability without correcting for mean values (using absolute SD values) showed lower variability for SHR/NCrl compared to WKY/NHsd rats for maximum velocity, with values that were 76, 69 and 73% of the corresponding values for the WKY/NHsd rats in the three sequences. For RMSSD, the absolute values were also lower for SHR/NCrl compared to WKY/NHsd rats, with values that were 78, 75 and 77% of the corresponding values for the WKY/NHsd rats in the three sequences (Table 4).

**Table 3:** Maximum velocity

	WKY	SHR	WKY	SHR	WKY	SHR
	0–28 min		28–56 min		56–84 min	
Mean	1302 ± 185	1455 ± 174**	1133 ± 206	1391 ± 166***	1137 ± 203	1388 ± 194***
SD	49 ± 8	33 ± 7***	56 ± 9	31 ± 5***	56 ± 12	33 ± 4***
RMSSD	62 ± 8	43 ± 8***	71 ± 11	43 ± 8***	72 ± 15	45 ± 7***
Sample entropy	1.94 ± 0.24	1.90 ± 0.21	2.01 ± 0.19	2.02 ± 0.19	1.96 ± 0.49	2.13 ± 0.27
Fourier analysis	0.66 ± 0.16	0.82 ± 0.18**	0.52 ± 0.09	0.75 ± 0.15***	0.55 ± 0.13	0.76 ± 0.22**
Symbolic dynamics						
0 V	0.1 ± 0.4	0.0 ± 0.0	0.3 ± 0.8	0.0 ± 0.0	1.2 ± 3.5	0.0 ± 0.0
1 V	1.8 ± 1.8	0.7 ± 0.9*	2.3 ± 3.0	0.8 ± 1.2*	3.7 ± 4.3	0.9 ± 1.0*
2LV	33.6 ± 2.9	34.2 ± 3.4	34.4 ± 3.3	32.3 ± 3.9	35.1 ± 4.7	33.7 ± 2.4
2UV	64.6 ± 3.4	65.1 ± 3.6	63.0 ± 3.4	66.9 ± 4.1**	60.0 ± 6.3	65.5 ± 2.5**

Maximum velocity was analyzed using time periods of 12.9 s. SD and RMSSD are given as % of the mean. For the Fourier analysis results are presented as variance in the high frequency range divided by the variance in the low frequency range. All data are given as mean ± SD

*t* tests: * p < 0.05, ** p < 0.01, *** p < 0.001

**Table 4:** Results from analysis of variability without correcting for mean values, but using absolute values for SD and RMSSD

	Motor activity			Mean velocity			Maximum velocity		
	WKY	SHR	p	WKY	SHR	p	WKY	SHR	p
SD									
0–28 min	880	1882	0.001	73	75	0.591	633	484	0.001
28–56 min	506	1438	0.001	70	74	0.452	631	435	0.001
56–84 min	481	1325	0.001	69	72	0.353	630	461	0.001
RMSSD									
0–28 min	2308	1021	0.001	86	93	0.095	813	634	0.001
28–56 min	1788	641	0.001	85	94	0.042	801	602	0.001
56–84 min	1702	629	0.001	84	95	0.016	809	624	0.001

Again, and similar to the findings for the mean velocity, the Fourier analysis showed that the SHR/NCrl rats had an increased ratio of variance in the high frequency range compared to the low frequency range. In SHR/NCrl, these were found to be 24, 44 and 38% higher than for the WKY/NHsd rats. The sample entropy values did not differ between the SHR/NCrl and the WKY/NHsd rats. The symbolic dynamic analyses showed that the SHR/NCrl rats had significantly lower values for the 1 V measure in all three sequences, and correspondingly higher values for 2UV in the second and third sequence.

In Table 5 are presented correlations between mean values of motor activity, mean velocity, maximum velocity and the different variability measures we have used in Tables 1, 2 and 3. These correlations are given for each strain separately and together. Analysis of sex differences did not reveal any consistent pattern with regard to differences between SHR/NCrl and WKY/NHsd rats, test sequences or the different parameters used, and are therefore not reported.

**Table 5:** Correlations between motor activity, mean velocity, maximum velocity and measures of variability in WKY and SHR rats in sequence 2 (28–56 min), each strain analyzed separately (A) and together (B)

		Motor activity		Mean velocity			Maximum velocity	
		WKY	SHR	WKY	SHR		WKY	SHR
*A*								
SD		−0.391	−0.523*	−0.691**	−0.114		−0.009	0.253
RMSSD		−0.611**	−0.596**	−0.821***	−0.443*		−0.091	0.171
Sample entropy		0.195	0.428*	−0.086	−0.382		0.653**	0.030
Fourier analysis		0.092	−0.328	0.250	−0.417*		0.315	−0.165
*Symbolic dynamics*								
0 V		−0.439	–	−0.207	–		0.115	–
1 V		−0.678**	−0.401	−0.211	−0.169		0.015	−0.417**
2LV		0.471*	0.016	0.278	0.018		−0.178	−0.112
2UV		0.437	0.026	−0.121	0.021		0.141	0.217

	Motor activity		Mean velocity		Maximum velocity	
*B*						
SD	−0.760***		−0.859***		−0.466**	
RMSSD	−0.802***		−0.875***		−0.472**	
Sample entropy	0.686***		−0.107		0.307*	
Fourier analysis	0.041		0.559***		0.407**	
*Symbolic dynamics*						
0 V	−0.505**		−0.283		−0.088	
1 V	−0.719***		−0.388*		−0.266	
2LV	0.559***		0.132		−0.272	
2UV	0.397**		0.102		0.396**	

## Discussion

The present study examined organization of video-recorded motor behavior in SHR/NCrl and WKY/NHsd controls using linear and non-linear methods. The main finding of the present study is that the motor activity of SHR/NCrl rats is different from WKY/NHsd rats in a number of ways, not only at the level of activity. The SHR/NCrl rats display increased mean and maximum velocity of their movements in addition to a pronounced increased total activity level. Concurrently, the organization of behavior is different in SHR/NCrl and WKY/NHsd controls. At a molar level of analysis, the variability of the time series, the SD and RMSSD, is markedly lower in SHR/NCrl compared to the WKY/NHsd rats when these measures are expressed as percent of the mean. At a molecular level of analysis, in contrast, the Fourier analysis shows that in the SHR/NCrl rats there is an increased variance in the high frequency part of the spectrum, corresponding to a time period of 9–17 s. When analyzing the time series with symbolic dynamics, the SHR/NCrl rats appear to have a higher behavioral complexity, particularly with regard to the total activity level. Similarly, using sample entropy, the complexity of the time series of total activity is higher in the SHR/NCrl rats than in the WKY/NHsd rats, and the lower autocorrelations of velocity in SHR/NCrl than in WKY/NHsd controls show that behavior is less systematic and less predictable from one occurrence to the next in the SHR/NCrl.

The increased total activity level of SHR/NCrl rats compared to the WKY/NHsd strain is in accordance with previous studies and in agreement with SHR/NCrl rats as a model of ADHD [20–24, 44, 45]. Increased activity is a defining feature of ADHD and has been confirmed using objective registrations of motor activity in patients [1, 46].

In SHR/NCrl, increased IIV has been found across a variety of behaviors including maze performance, lever pressing and nose poking [20–24, 44, 45]. The markedly reduced molar IIV in SHR/NCrl, as measured with SD and RMSSD, found in the present study is therefore at first glance surprising and inconsistent with the findings of Perry et al. [24] who used an identical experimental procedure to the one used in the present study, where total test-time was divided into 5 segments, and IIV for operant lever-pressing was expressed as the absolute difference between behavior in each segment and the total test-time mean. One important difference between the studies is that Perry et al. analyzed reinforcer-controlled lever pressing only, whereas the video-recorded behavior analyzed in the present study included reinforcer-controlled movements (lever approach, presses, tray visits, and reinforcer consummation) as well as other movements not controlled by the scheduled reinforcers (e.g. grooming, exploration and motor control). The impact of each of these processes on the observed changes in IIV in SHR/NCrl cannot be disentangled in the present study, but may have contributed to the inconsistent findings. A second important difference between the two studies is that Perry et al. used variability measures corrected for mean whereas SD and RMSSD mean corrections were used in the present study. Although uncorrected SDs and RMSSDs in the present study were higher in SHR/NCrl than in controls for total activity, the means were also much higher in SHR/NCrl than in controls. Thus, the mean-corrections produced lower SDs and RMSSDs in SHR/NCrl than in controls, and it has been argued that this procedure may be overly conservative and overcorrect for SHR/NCrl phenotype [24]. In the analysis of mean velocity, uncorrected SDs and RMSSDs were also higher in SHR/NCrl than in controls, but the differences were smaller, whereas uncorrected SDs and RMSSDs for maximum velocity were lower in SHR/NCrl than in controls. Comparing total activity, mean and maximum velocity using uncorrected SD and RMSSD would therefore give inconsistent results, while correcting for mean gives a consistent picture, with lower SD and RMSSD for SHR/NCrl compared to controls in the range of 51–69%.

Mean corrections have been discussed within the ADHD literature for measures of reaction time (RT) and reaction time variability. In these studies, intraindividual variability has commonly been measured as the standard deviation of RTs without mean correction. Studies have shown that although correlated, RT mean and RT standard deviation have independent components of variance [47]. Additionally, increased mean RT and RT variability may have shared etiology in ADHD [48]. Thus, by correcting for mean, there is a risk of controlling for what one intends to study [49].

The question of dependence between the mean and measures of variability is highly relevant in the present study because the increased mean activity level and variability measures in SHR/NCrl could be expressions of one underlying factor. When looking at data from both rat strains, there are strong correlations between the variability measures and mean values for motor activity, velocity and maximum velocity, and these correlations parallel the differences in variability measures between the strains. However, when examining each strain separately there are fewer correlations and the pattern is clearly different for the two strains. We think this shows that the differences seen between the two strains do not simply reflect differences in total motor activity or velocity of movement, and that studying variability measures give added information concerning the organization of motor activity.

Overall, the analyses of video-recorded behavior during the operant task suggest that behavior is organized differently in SHR/NCrl as compared to WKY/NHsd controls: At a molar level, SHR/NCrl behavior is less variable whereas behavior at a molecular level is more complex than in controls. Increased molecular behavioral complexity in SHR/NCrl compared to WKY/NHsd was found in the Fourier analyses for both mean velocity and maximum velocity of movement, and is consistent with the symbolic dynamics analyses, and the autocorrelations analyses for velocity of movement.

Studying movement patterns, Paulus et al. [50] found differences between Fischer, Lewis, and Sprague–Dawley rats using a spatial scaling exponent quantifying the degree of linear movement versus movement within a circumscribed area (low versus high scaling exponent, respectively), that may in some respect resemble the complexity test we have used. They suggested that a lower scaling exponent in Sprague–Dawley rats compared to Fischer and Lewis rats was related to differences in central serotonergic systems. In a study of SHR and WKY rats, Li and Huang [51] found that the scaling exponent was higher in SHR rats, in accordance with our finding of a higher complexity of total motor activity in these rats. Previous studies have shown a range of neurological changes in SHR. We are in our study unable to separate the possible role of dopaminergic and serotonergic systems in the regulation of movement patterns, and there are differences between SHR and WKY rats in both these systems. Additionally, changes in noradrenergic, glutaminergic neurotransmission and several other systems have been shown in SHR [19, 26, 52–55].

The present finding may partly reflect basic motor processes and point to important differences in the neuronal organization of basic motor activity in SHR/NCrl compared to WKY/NHsd rats. This may indicate similar differences in motor activity regulation in patients with ADHD vs. controls. In a study of reaction times during the CPT-II test, higher variability (using SD and RMSSD) was found in adult ADHD patients compared to clinical controls, but at the same time lower complexity as measured with sample entropy and symbolic dynamic analysis was found in the ADHD group [31]. This finding, an inverse relation between measures of variability and complexity, mirrors the relation between the same measures in the present study. We have seen this same inverse relationship also in a study of motor activity in depressed and schizophrenic patients [32].

Reduced complexity of physiological systems has been postulated to be associated with disease and aging [28], but this may depend on the dynamics of the system under study. Vaillancourt and Newell [56] have suggested that in systems with intrinsic oscillations the opposite may occur, namely that disease processes are accompanied by increased complexity. This has been found in the motor activity of schizophrenic patients [32], and the present findings may fit the same pattern.

Another way to conceptualize the present findings on intraindividual variability is to compare them with human studies showing that variability patterns are different when comparing measures of brain function and behavior. Garrett et al. [57] found in an imaging study that blood oxygen level-dependent signal variability (brain variability) was lower in older compared to younger persons, while reaction time speed variability on different cognitive tasks was higher. Similarly, McIntosh et al. [58] found, when comparing children and young adults, that maturation was accompanied by increased variability of EEG-signals and reduced variability of response times on a facial recognition task.

Studies of behavioral variability in ADHD have produced a complex set of findings. Studying children with ADHD using autocorrelations, predictability of responses was found to be lower in ADHD (i.e. responding was more variable), consistent with the current findings [2]. Additionally, the autocorrelations in ADHD were found to be sensitive to the reinforcement contingencies [3], which has also been found for response time variability [59]. In a study of reaction times in children with ADHD, Castellanos et al. [6] found evidence of multisecond oscillations, with a cycle length of approximately 20 s, and they suggested that this might be due to deficiencies in dopaminergic regulations in the patients. This is intriguingly similar to the findings with Fourier analysis in the present study. Using Fourier analyses, Karalunas et al. found more low-frequency variability and higher faster-frequency variability in ADHD, with non-significant differences between frequency bands [60]. In a study of children with ADHD, Wood et al. [46] found, in addition to increased motor activity, also increased intraindividual variability of the intensity of movements. On the other hand [61], a study of adult ADHD patients found that the patients had both increased activity levels and reduced daytime variability patterns compared to controls. In another study in adults, ADHD patients did not show increased activity levels compared to controls, and variability measures (SD and RMSSD) were not altered, but Fourier analyses revealed higher power in the high frequency range, corresponding to the period from 2 to 8 min [31].

Several mechanisms underlying the increased IIV observed in ADHD have been proposed, including deficient astrocyte energy supply to active neurons, state regulation and working memory problems, arousal-attention regulation, and altered learning processes (see [11, 49] for reviews of etiological models of reaction time variability). The complexity of findings is a challenge to current theories of IIV in ADHD, and obviously underscore the need for further studies that compare measures used to characterize variability, examine possible discrepancies between molar and molecular analyses of variability, and explore variability patterns in both patients and animal models.

The current findings add to this complexity by suggesting the presence of both increased molecular as well as decreased molar behavioral variability in SHR. If valid for ADHD, this finding is a new and interesting contribution to the research on IIV, and suggests that IIV in ADHD is not unitary and explained by one common principle, but may have several underlying mechanism depending on the task used and the behavior analyzed, and may be changed in opposite directions depending on the variability measures used.

There are some important limitations to the present study that must be considered. First, it is not clear what the video-recorded behavior during the operant task reflect (i.e. reinforcer-effects, grooming, exploration, basic motor organization, or other processes) or how the behavioral changes relate to underlying mechanisms. Nevertheless, several changes in IIV in SHR/NCrl were found suggesting that analyses of video-recorded behavior may be a valuable supplement to traditional behavioral measures used in studies of IIV. Second, the decreased molar IIV found in SHR/NCrl relative to controls is based on analyses of SD and RMSSD correcting for mean. However, the use of mean correction has been debated in the ADHD literature, and has been argued to overcorrect for phenotype in studies of SHR/NCrl [24]. The present analyses using mean corrections produced more consistent results, with variability changes in opposite directions, compared to analyses using mean corrections, underscoring the importance of mean corrections in analyses of variability.

## Conclusion

This study shows that SHR/NCrl rats, a postulated animal model of ADHD, are different form WKY/NHsd rats in a number of measures related to motor activity. In addition to increased activity levels, the most pronounced findings are increased mean and maximum velocity of movements, and reduced variability for all these measures when assessed with SD and RMSSD corrected for mean. There is also an increased complexity of movement patterns in the SHR/NCrl rats. These results point to differences in the neuronal organization of movements that may be related to the known differences in neurotransmitter systems between these two rat strains. Even though these findings have no immediate implications for the diagnosis or treatment of ADHD patients, they may be used to explore further the mechanisms of motor activity regulation in general, and alterations in neurodevelopmental disorders such as ADHD.

## Abbreviations

ADHD: attention-deficit/hyperactivity disorder
ANOVA: analysis of variance
EEG: electroencephalogram
IIV: intra-individual variability
PND: post-natal day
RMSSD: root mean square successive difference (the square root of the mean of the squares of the differences between adjacent time periods)
SD: standard deviation
SHR/NCrl: spontaneously hypertensive rats bred by Charles River (an animal model of ADHD)
WKY/NHsd: Wistar Kyoto rats bred by Harlan (a control animal for the SHR)
0V: symbolic dynamic measure representing a pattern with no variation (e.g. pattern 333 or 555)
1V: symbolic dynamic measure representing a pattern with only one variation (two consecutive symbols are equal and the remaining symbol is different, e.g. 522 or 331)
2LV: symbolic dynamic measure representing a pattern with two like variations (the 3 symbols ascend or descend, e.g., 641 or 235)
2UV: symbolic dynamic measure representing a patterns with two unlike variations (both ascending and descending, e.g., 312 or 451)

## References

[1] Teicher MH (1995). Actigraphy and motion analysis: new tools for psychiatry. Harv Rev Psychiatry.

[2] Aase H, Meyer A, Sagvolden T (2006). Moment-to-moment dynamics of ADHD behaviour in South African children. Behav Brain Funct.

[3] Aase H, Sagvolden T (2005). Moment-to-moment dynamics of ADHD behaviour. Behav Brain Funct.

[4] Adamo N, Huo L, Adelsberg S, Petkova E, Castellanos FX, Di Martino A (2014). Response time intra-subject variability: commonalities between children with autism spectrum disorders and children with ADHD. Eur Child Adolesc Psychiatry.

[5] Castellanos FX, Kelly C, Milham MP (2009). The restless brain: attention-deficit hyperactivity disorder, resting-state functional connectivity, and intrasubject variability. Can J Psychiatry.

[6] Castellanos FX, Sonuga-Barke EJ, Scheres A, Di Martino A, Hyde C, Walters JR (2005). Varieties of attention-deficit/hyperactivity disorder-related intra-individual variability. Biol Psychiatry.

[7] Di Martino A, Ghaffari M, Curchack J, Reiss P, Hyde C, Vannucci M, Petkova E, Klein DF, Castellanos FX (2008). Decomposing intra-subject variability in children with attention-deficit/hyperactivity disorder. Biol Psychiatry.

[8] Johnson KA, Kelly SP, Bellgrove MA, Barry E, Cox M, Gill M, Robertson IH (2007). Response variability in attention deficit hyperactivity disorder: evidence for neuropsychological heterogeneity. Neuropsychologia.

[9] Karalunas SL, Geurts HM, Konrad K, Bender S, Nigg JT (2014). Annual research review: reaction time variability in ADHD and autism spectrum disorders: measurement and mechanisms of a proposed trans-diagnostic phenotype. J Child Psychol Psychiatry.

[10] Klein C, Wendling K, Huettner P, Ruder H, Peper M (2006). Intra-subject variability in attention-deficit hyperactivity disorder. Biol Psychiatry.

[11] Kofler MJ, Rapport MD, Sarver DE, Raiker JS, Orban SA, Friedman LM, Kolomeyer EG (2013). Reaction time variability in ADHD: a meta-analytic review of 319 studies. Clin Psychol Rev.

[12] Luman M, Papanikolau A, Oosterlaan J (2015). The unique and combined effects of reinforcement and methylphenidate on temporal information processing in attention-deficit/hyperactivity disorder. J Clin Psychopharmacol.

[13] Russell VA, Oades RD, Tannock R, Killeen PR, Auerbach JG, Johansen EB, Sagvolden T (2006). Response variability in attention-deficit/hyperactivity disorder: a neuronal and glial energetics hypothesis. Behav Brain Funct.

[14] Sagvolden T, Aase H, Zeiner P, Berger D (1998). Altered reinforcement mechanisms in attention-deficit/hyperactivity disorder. Behav Brain Res.

[15] Shiels Rosch K, Dirlikov B, Mostofsky SH (2013). Increased intrasubject variability in boys with ADHD across tests of motor and cognitive control. J Abnorm Child Psychol.

[16] Tamm L, Narad ME, Antonini TN, O’Brien KM, Hawk LW, Epstein JN (2012). Reaction time variability in ADHD: a review. Neurotherapeutics.

[17] van Belle J, van Hulst BM, Durston S (2015). Developmental differences in intra-individual variability in children with ADHD and ASD. J Child Psychol Psychiatry.

[18] Williams BR, Strauss EH, Hultsch DF, Hunter MA, Tannock R (2007). Reaction time performance in adolescents with attention deficit/hyperactivity disorder: evidence of inconsistency in the fast and slow portions of the RT distribution. J Clin Exp Neuropsychol.

[19] Sagvolden T, Johansen EB (2012). Rat models of ADHD. Curr Top Behav Neurosci.

[20] Hunziker MH, Saldana RL, Neuringer A (1996). Behavioral variability in SHR and WKY rats as a function of rearing environment and reinforcement contingency. J Exp Anal Behav.

[21] Johansen EB, Killeen PR, Sagvolden T (2007). Behavioral variability, elimination of responses, and delay-of-reinforcement gradients in SHR and WKY rats. Behav Brain Funct.

[22] Mook DM, Jeffrey J, Neuringer A (1993). Spontaneously hypertensive rats (SHR) readily learn to vary but not repeat instrumental responses. Behav Neural Biol.

[23] Perry GM, Sagvolden T, Faraone SV (2010). Intra-individual variability in genetic and environmental models of attention-deficit/hyperactivity disorder. Am J Med Genet B Neuropsychiatr Genet.

[24] Perry GM, Sagvolden T, Faraone SV (2010). Intraindividual variability (IIV) in an animal model of ADHD– the spontaneously hypertensive Rat. Behav Brain Funct.

[25] van den Bergh FS, Bloemarts E, Chan JS, Groenink L, Olivier B, Oosting RS (2006). Spontaneously hypertensive rats do not predict symptoms of attention-deficit hyperactivity disorder. Pharmacol Biochem Behav.

[26] Sagvolden T, Johansen EB, Woien G, Walaas SI, Storm-Mathisen J, Bergersen LH, Hvalby O, Jensen V, Aase H, Russell VA (2009). The spontaneously hypertensive rat model of ADHD—the importance of selecting the appropriate reference strain. Neuropharmacology.

[27] Goldberger AL (1996). Non-linear dynamics for clinicians: chaos theory, fractals, and complexity at the bedside. Lancet.

[28] Goldberger AL (1997). Fractal variability versus pathologic periodicity: complexity loss and stereotypy in disease. Perspect Biol Med.

[29] Tang L, Lv H, Yang F, Yu L (2015). Complexity testing techniques for time series data: a comprehensive literature review. Chaos Solitons Fractals.

[30] Shimp CP (2014). What means in molecular, molar, and unified analyses. Int J Comp Psychol.

[31] Fasmer OB, Mjeldheim K, Forland W, Hansen AL, Syrstad VE, Oedegaard KJ, Berle JO (2016). Linear and non-linear analyses of Conner’s continuous performance test-II discriminate adult patients with attention deficit hyperactivity disorder from patients with mood and anxiety disorders. BMC Psychiatry.

[32] Hauge ER, Berle JO, Oedegaard KJ, Holsten F, Fasmer OB (2011). Nonlinear analysis of motor activity shows differences between schizophrenia and depression: a study using Fourier analysis and sample entropy. PLoS ONE.

[33] Krane-Gartiser K, Henriksen TE, Morken G, Vaaler A, Fasmer OB (2014). Actigraphic assessment of motor activity in acutely admitted inpatients with bipolar disorder. PLoS ONE.

[34] Fasmer OB, Hauge E, Berle JO, Dilsaver S, Oedegaard KJ (2016). Distribution of active and resting periods in the motor activity of patients with depression and Schizophrenia. Psychiatry Investig.

[35] Johansen EB, Fonnum F, Lausund PL, Walaas SI, Baerland NE, Woien G, Sagvolden T (2014). Behavioral changes following PCB 153 exposure in the spontaneously hypertensive rat-an animal model of attention-deficit/hyperactivity disorder. Behav Brain Funct.

[36] Sagvolden T, Xu T (2008). l-Amphetamine improves poor sustained attention while d-amphetamine reduces overactivity and impulsiveness as well as improves sustained attention in an animal model of attention-deficit/hyperactivity disorder (ADHD). Behav Brain Funct.

[37] Jensenius AR, Godøy RI, Wanderley MM. Developing tools for studying musical gestures within the Max/MSP/Jitter environment. In: Proceedings of the International Computer Music Conference. 2005: 282–5.

[38] Jensenius AR (2007). Action-sound: developing methods and tools to study music-related body movement.

[39] Fasmer OB, Liao H, Huang Y, Berle JO, Wu J, Oedegaard KJ, Wik G, Zhang Z (2012). A naturalistic study of the effect of acupuncture on heart-rate variability. J Acupunct Meridian Stud.

[40] Goldberger AL, Amaral LA, Glass L, Hausdorff JM, Ivanov PC, Mark RG, Mietus JE, Moody GB, Peng CK, Stanley HE (2000). PhysioBank, PhysioToolkit, and PhysioNet: components of a new research resource for complex physiologic signals. Circulation.

[41] Richman JS, Moorman JR (2000). Physiological time-series analysis using approximate entropy and sample entropy. Am J Physiol Heart Circ Physiol.

[42] Guzzetti S, Borroni E, Garbelli PE, Ceriani E, Della Bella P, Montano N, Cogliati C, Somers VK, Malliani A, Porta A (2005). Symbolic dynamics of heart rate variability: a probe to investigate cardiac autonomic modulation. Circulation.

[43] Porta A, Tobaldini E, Guzzetti S, Furlan R, Montano N, Gnecchi-Ruscone T (2007). Assessment of cardiac autonomic modulation during graded head-up tilt by symbolic analysis of heart rate variability. Am J Physiol Heart Circ Physiol.

[44] Low WC, Whitehorn D, Hendley ED (1984). Genetically related rats with differences in hippocampal uptake of norepinephrine and maze performance. Brain Res Bull.

[45] Mook DM, Neuringer A (1994). Different effects of amphetamine on reinforced variations versus repetitions in spontaneously hypertensive rats (SHR). Physiol Behav.

[46] Wood AC, Asherson P, Rijsdijk F, Kuntsi J (2009). Is overactivity a core feature in ADHD? Familial and receiver operating characteristic curve analysis of mechanically assessed activity level. J Am Acad Child Adolesc Psychiatry.

[47] Jensen AR (1992). The importance of intraindividual variation in reaction time. Personal Individ Differ.

[48] McLoughlin G, Palmer JA, Rijsdijk F, Makeig S (2014). Genetic overlap between evoked frontocentral theta-band phase variability, reaction time variability, and attention-deficit/hyperactivity disorder symptoms in a twin study. Biol Psychiatry.

[49] Kuntsi J, Klein C. Intraindividual variability in ADHD and its implications for research of causal links. In: Behavioral neuroscience of attention deficit hyperactivity disorder and its treatment. Berlin: Springer; 2011. p. 67–91. 10.1007/7854_2011_145 21769722

[50] Paulus MP, Geyer MA, Sternberg E (1998). Differential movement patterns but not amount of activity in unconditioned motor behavior of Fischer, Lewis, and Sprague-Dawley rats. Physiol Behav.

[51] Li JS, Huang YC (2006). Early androgen treatment influences the pattern and amount of locomotion activity differently and sexually differentially in an animal model of ADHD. Behav Brain Res.

[52] Dervola KS, Roberg BA, Woien G, Bogen IL, Sandvik TH, Sagvolden T, Drevon CA, Johansen EB, Walaas SI (2012). Marine Omicron-3 polyunsaturated fatty acids induce sex-specific changes in reinforcer-controlled behaviour and neurotransmitter metabolism in a spontaneously hypertensive rat model of ADHD. Behav Brain Funct.

[53] Russell VA (2003). Dopamine hypofunction possibly results from a defect in glutamate-stimulated release of dopamine in the nucleus accumbens shell of a rat model for attention deficit hyperactivity disorder—the spontaneously hypertensive rat. Neurosci Biobehav Rev.

[54] Russell VA, Sagvolden T, Johansen EB (2005). Animal models of attention-deficit hyperactivity disorder. Behav Brain Funct.

[55] Sagvolden T, Russell VA, Aase H, Johansen EB, Farshbaf M (2005). Rodent models of attention-deficit/hyperactivity disorder. Biol Psychiatry.

[56] Vaillancourt DE, Newell KM (2002). Changing complexity in human behavior and physiology through aging and disease. Neurobiol Aging.

[57] Garrett DD, Kovacevic N, McIntosh AR, Grady CL (2011). The importance of being variable. J Neurosci.

[58] McIntosh AR, Kovacevic N, Itier RJ (2008). Increased brain signal variability accompanies lower behavioral variability in development. PLoS Comput Biol.

[59] Tye C, Johnson KA, Kelly SP, Asherson P, Kuntsi J, Ashwood KL, Azadi B, Bolton P, McLoughlin G (2016). Response time variability under slow and fast-incentive conditions in children with ASD, ADHD and ASD+ADHD. J Child Psychol Psychiatry..

[60] Karalunas SL, Huang-Pollock CL, Nigg JT (2013). Is reaction time variability in ADHD mainly at low frequencies?. J Child Psychol Psychiatry.

[61] Boonstra AM, Kooij JJ, Oosterlaan J, Sergeant JA, Buitelaar JK, Van Someren EJ (2007). Hyperactive night and day? Actigraphy studies in adult ADHD: a baseline comparison and the effect of methylphenidate. Sleep.

